# Identification of temporal association rules from time-series microarray data sets

**DOI:** 10.1186/1471-2105-10-S3-S6

**Published:** 2009-03-19

**Authors:** Hojung Nam, KiYoung Lee, Doheon Lee

**Affiliations:** 1 Department of Bio and Brain Engineering, KAIST, 373-1 Guseong-dong, Yuseong-gu, Daejeon, Korea; 2 Department of Bioengineering University of California at San Diego, La Jolla, California 92093, USA

## Abstract

**Background:**

One of the most challenging problems in mining gene expression data is to identify how the expression of any particular gene affects the expression of other genes. To elucidate the relationships between genes, an association rule mining (ARM) method has been applied to microarray gene expression data. However, a conventional ARM method has a limit on extracting temporal dependencies between gene expressions, though the temporal information is indispensable to discover underlying regulation mechanisms in biological pathways. In this paper, we propose a novel method, referred to as temporal association rule mining (TARM), which can extract temporal dependencies among related genes. A temporal association rule has the form [*gene A*↑, *gene B*↓] → (7 min) [*gene C*↑], which represents that high expression level of *gene A *and significant repression of *gene B *followed by significant expression of *gene C *after 7 minutes. The proposed TARM method is tested with *Saccharomyces cerevisiae *cell cycle time-series microarray gene expression data set.

**Results:**

In the parameter fitting phase of TARM, the fitted parameter set [threshold = ± 0.8, support ≥ 3 transactions, confidence ≥ 90%] with the best precision score for KEGG cell cycle pathway has been chosen for rule mining phase. With the fitted parameter set, numbers of temporal association rules with five transcriptional time delays (0, 7, 14, 21, 28 minutes) are extracted from gene expression data of 799 genes, which are pre-identified cell cycle relevant genes. From the extracted temporal association rules, associated genes, which play same role of biological processes within short transcriptional time delay and some temporal dependencies between genes with specific biological processes are identified.

**Conclusion:**

In this work, we proposed TARM, which is an applied form of conventional ARM. TARM showed higher precision score than Dynamic Bayesian network and Bayesian network. Advantages of TARM are that it tells us the size of transcriptional time delay between associated genes, activation and inhibition relationship between genes, and sets of co-regulators.

## Background

The genome of an organism plays a central role in the control of cellular processes such as genetic regulation, metabolic pathway, and signal transduction. Because these processes are very complex and comprised of many genetic interacting elements, it is hard to discover those interacting elements in the complex biological regulations. Since microarray technique allows researchers to simultaneously observe the expression levels of thousands of genes in a single experiment, there have been many studies to discover global genetic regulation from microarray gene expression data by using various computational methods to uncover the hidden roles of genetic elements, such as clustering techniques to identify clusters of co-expressed genes [1-3], network inference techniques to construct the genome-wide regulatory network models [4-9].

One of the most challenging problems in analyzing gene expression data is to determine how the expression of any particular gene might affect the expression of other genes. To find the relationships among different genes, an association rule mining (ARM) method has been applied to gene expression data set because the method can identify associations among genes even when the genes are not co-expressed [10-14]. An association rule has the form *LHS *(Left Hand Side) → *RHS *(Right Hand Side), where *LHS *and *RHS *are sets of items, and it represents that the *RHS *set being likely to occur whenever the *LHS *set occurs. In case of analyzing gene expression data, the items in an association rules are represented as genes, which are highly expressed or highly repressed. An example of an association rule from gene expression data might be [*gene A*↑, *gene B*↓] → [*gene C*↑], which represents that when *gene A *is measured as highly expressed and *gene B *is highly repressed then it is also likely to observe and *gene C *is highly expressed. From the result of the ARM method, it is possible to discover interactions between correlated expressions of genes in microarray experiments. Despite of the usefulness of ARM [12], the time dependency between associated genes cannot be extracted by using the conventional ARM method even though the temporal information is indispensable to discover regulation mechanisms.

Previous studies, which identify time-dependent regulatory relations among genes can be grouped into two general categories. The first approach constructs cellular dynamic models to observe the response of cells by using dynamic Bayesian network (DBN) [15-18] and ordinary differential equation (ODE). However, these approaches have fundamental problems: They need a huge amount of computational time to infer the temporal dependency among genes and show relatively low accuracies analyzing in microarray gene expression data [16,18]. These drawbacks are mainly caused by the fact that the currently available time-series microarray data is not suited for such complex models of genetic regulation. Most of microarray gene expression data sets have relatively small number of experiments compared to the number of genes and they have relatively large regular time intervals between experiment time points. The second approach identifies pair-wise temporal dependency between genes by clustering with local patterns of gene expression [19], by measuring the Pearson correlation coefficient of two genes, by detecting the major changes in expression level [20], by scoring the expression patterns with several defined events [21], and by matching the expression patterns with shifted patterns [2,3]. Although such methods can identify pair-wise temporal relations, it cannot identify combinatorial temporal relations which are regarded an important characteristic of regulation [22,23]. For example, the meaning of [*gene A*, *gene B*] → (7 min) [*gene C*], and [*gene A*] → (7 min) [*gene C*] 'AND' [*gene B*] → (7 min) [*gene C*] is completely different: In the case of [*gene A*, *gene B*] → (7 min) [*gene C*], *gene A *and *gene B *play a role as combinatorial regulators in a single regulation. On the other hand, [*gene A*] → (7 min) [*gene C*] AND [*gene B*] → (7 min) [*gene C*], *gene A *and *gene B *are independent regulators.

Even though there are some previous studies related to extraction of association rules from time series data in other application domains [24,25], they do not provide temporal dependencies among items within different time (e.g. time shifted, time delayed). To address the problem, we propose a new mining method for gene expression data sets, which can extract temporal dependency among genes by applying temporal association rule mining (TARM) method. The temporal association rules represent various transcriptional time delays between associated genes. An example of a temporal association rule is [*gene A*↑, *gene B*↓] → (7 min) [*gene C*↑], which represents that high expression level of *gene A *and significant repression of *gene B *followed by significant expression of *gene C *after 7 minutes. Hence, the temporal association rule can tell us the size of transcriptional time delay (7 minutes) between associated genes (*gene A, gene B and gene C*), activation and inhibition relationship (*gene A*↑ → *gene C*↑), and sets of co-regulators (*gene A*↑, *gene B*↓).

**Figure 1:**
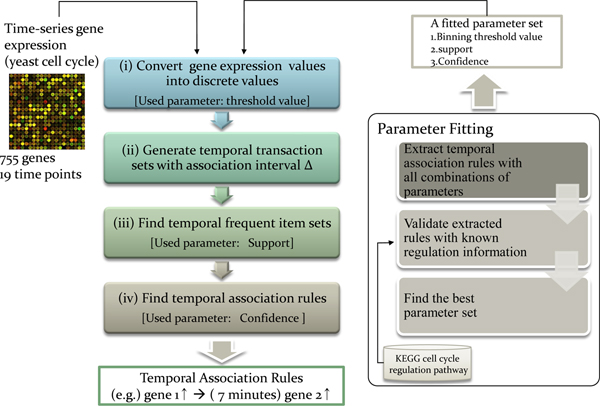
**Method overview**. (a) The overall phase of proposed method. (b) Parameter fitting phase.

The overall process of the proposed method is depicted in Figure 1. The proposed method consists of two main phases. First, temporal association rule mining phase. With an obtained fitted parameter set, the steps of temporal association mining method is applied to time-series gene expression data: (i) converting gene expression values into discrete values, (ii) generating temporal transaction sets with various sizes of transcriptional time delay Δ, (iii) generating temporal frequent item sets, (iv) and finally, extracting temporal association rules. The proposed method is tested with public microarray experiments of *Saccharomyces cerevisiae *cell cycle alpha factor arrest synchronization data set. Second, parameters fitting phase. In this phase, external known regulation information (KEGG cell cycle regulation information) is used to choose the best parameter set from all possible combinations of parameter sets. Three parameters are selected for the proposed temporal association rule mining (TARM) method. Among every possible combination of three parameter values, the best parameter set that has the highest overlap degree with previously known biological regulation relationships is selected as the fitted parameter set.

## Methods

### Conventional association rule mining (Apriori algorithm)

To explain the basic concepts of association rule mining, we use the definitions and the examples of supermarket data shown in [26]. Consider a small store that sells the following set of items: [Bagels, Bread, Butter, Cereal, Juice, Milk]. List of items bought by six hypothetical customers are shown in Table 1. This table will be used to illustrate the concepts presented in this section.

**Table 1:** List of items bought by six customers. Each row of the table is referred to as a transaction.

**No**.	**Item purchased**
1	Bread, Butter, Cereal, Juice, Milk
2	Cereal, Juice, Milk
3	Bagels, Butter, Cereal, Juice, Milk
4	Bread, Cereal, Jelly, Juice, Milk
5	Bagels, Jelly, Juice, Milk
6	Jelly, Juice, Milk

#### Definition 1

(1) An **association rule **is a pair of disjoint item sets. If *LHS *(Left Hand Side) and *RHS *(Right Hand Side) denote the two disjoint item sets, the association rule is written as *LHS *→ *RHS*.

(2) The **support **of the association rule *LHS *→ *RHS *with respect to a transaction set *T *is the support of the item set *LHS ∪ RHS *with respect to *T*.

(3) The **confidence **of the rule *LHS *→ *RHS *with respect to a transaction set *T *is the ratio support (*LHS *→ *RHS)*/support(*LHS*).

#### Example

Consider the item sets A_1 _= [Juice, Milk] and A_2 _= [Cereal]. Since A_1 _and A_2 _are disjoint, A_1 _→ A_2 _(or equivalently, [Juice, Milk] → [Cereal]) is an association rule. Let R_1 _denote this association rule. The support of R_1 _is the support of the item set [Juice, Milk, Cereal]. From Table 1, it can be seen that this support value is 4. Also from Table 1, the support of the item set [Juice, Milk] is 6. Therefore, the confidence of Rule R_1 _is 4/6 or 66.67%.

### Temporal association rule mining (TARM)

In this work, we propose a temporal association rule mining (TARM) method, which is based on Apriori algorithm. Following two sub-sections will explain the detailed methodology of temporal association rule mining phase (Figure 1(b)), and parameter fitting phase (Figure 1(a)).

To explain the concept of the proposed TARM method, we first define new terminologies.

#### Definition 2

(1) A **temporal item **is an item, which has a time stamp.

(2) A **temporal item set **Ï is a non-empty set of temporal items.

(3) Given a temporal item set Ï, a set T of transactions on Ï, and a positive integer α, Ï is a temporal frequent item set with respect to T and α if **support **T (Ï) > = α. (α is the support threshold.)

(4) A **temporal association rule **is a pair of disjoint temporal item sets. If *LHS *and *RHS *denote the left and right temporal item sets respectively, then the time stamp of each temporal item in LHS is ahead of those of all temporal items in *RHS*. A temporal association rule is written as *LHS *> (Δ) *RHS*, where Δ is the interval of different two time stamps.

**Figure 2:**
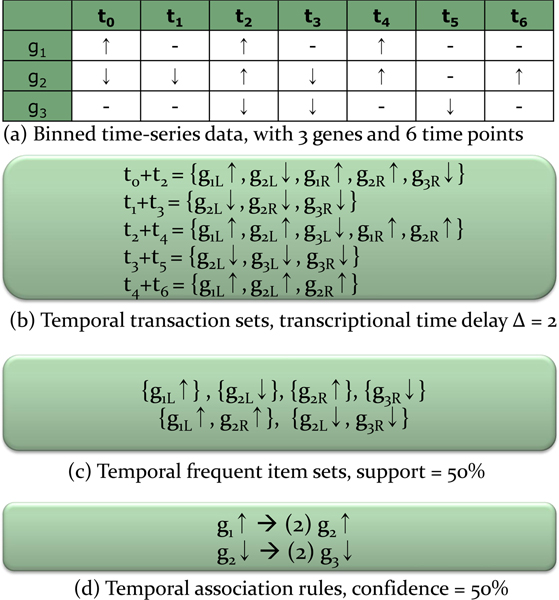
**An illustration of temporal association rule mining process**. An illustration of temporal association rule mining process with transcriptional time delay Δ = 2, support ≥ 50%, confidence ≥ 50%.

Figure 2 shows an illustration of temporal association rule mining process. First, continuous gene expression values are converted into discrete values (up, down, and none) (Figure 2(a)). Second, to find temporally associated genes, we first assume that all related genes may have various sizes of transcriptional time delay. Therefore, our method searches associated genes in all possible sets of different time point experiments where the time interval is from 0 to *n *(Figure 2(b)). In this illustration, Δ is 2. For example, Temporal transaction set t_0 _+ t_2 _= [g_1*L*_↑, g_2*L*_↓, g_1*R*_↑, g_2*R*_↑, g_3*R*_↓] consists of up or down regulated genes at time stamps t_0 _and t_2 _with the size of transcriptional time delay Δ = 2. Note that, for g_1_, it is up regulated in both cases of t_0 _and t_2_, but we marked them as two different genes like g_1*L *_(g_1 _in Left hand side) and g_1*R *_(g_1 _in Right hand side). Third, Figure 2(c) indicates the extracted temporal frequent item sets with support threshold 50%. And finally, two temporal association rules are discovered with confidence threshold 50% as shown in Figure 2(d). In this manner, TARM can find (1) various sizes of transcriptional time delay between associated genes, (2) activation and inhibition relationship, (3) sets of co-regulators for the target genes.

### Parameter extraction

This section shows the phase for obtaining three different parameters which are necessary when mining temporal association rules: (1) a cutoff value for binning transcriptional expression values, (2) a support value for mining temporal frequent item sets, and (3) a confidence value for extracting temporal association rules. Since the performance of the proposed method is dependent on the parameter set, the parameter set should be chosen very carefully. If the ground truths of cell cycle regulation are known, the regulation information can be used to fit the parameters. However, absence of such kinds of information, alternative information source is used. In this study, we utilize KEGG cell cycle regulation path as known information set to find the best parameter set which can extract the most number of accurate temporal association rules. The KEGG cell cycle regulation path is a collection of manually drawn pathway maps representing the regulation knowledge on the molecular interaction, and the pathway contains interaction information which are relevant to cell cycle of yeast [27,28].

The KEGG regulation information is used for a measure of correctness of the extracted candidate rules with various combinations of parameters. If an extracted temporal association rule is matched with KEGG regulation information, then we regard the rule as a correctly extracted rule. Namely, the validation score is calculated by the following equation:

(1) $$precision=\frac{(\#of\;matchedrules\text{)}}{\left(\#ofextractedrules\right)}$$

To select a fitted parameter set among the various combinations, we select a parameter set which shows the highest validation score.

## Results and discussion

### Data sets

To check the performance of the proposed method, we used *S. cerevisiae *cell cycle alpha factor arrest synchronization microarray data set [29]. This time-series microarray data set has 18 time points with relatively small regular time intervals (7 minutes) between every sampling time point.

### Fitted parameters

**Figure 3:**
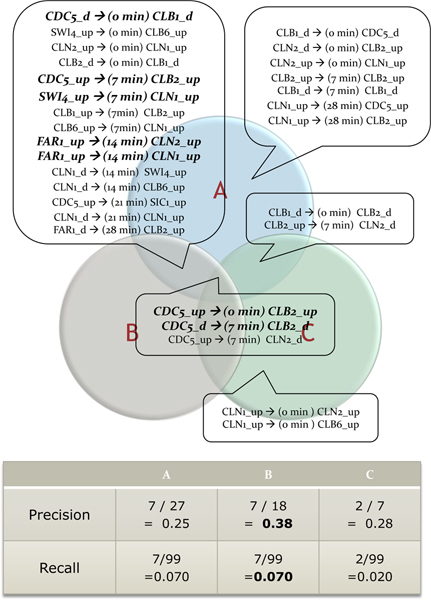
**Extracted temporal association rules with the selected three parameter sets**. Best three parameter sets are selected to compare results of extracted rules on cell cycle expression data of 57 genes with association delay 0 ~28 minutes. Set A = [threshold = ± 0.8, support ≥ 3 transactions, confidence ≥ 80%], set B = [threshold = ± 0.8, support ≥ 3 transactions, confidence ≥ 90%], set C = [threshold = ± 1.0, support ≥ 3 transactions, confidence ≥ 80%]. The intersection area of a Venn diagram stands for the commonly extracted rules with different parameter sets. Rules written in Italic font denote known regulation relations in KEGG Cell cycle pathway data.

In the parameter fitting phase, combination sets of parameters are generated within binning cutoff values from 0.2 to 1.4, support cutoff values from 2 to 6 transaction, and confidence cutoff values from 80 to 100%. With these parameter sets, TARM method is applied on cell cycle expression data of 57 genes, which are nodes of KEGG yeast cell cycle regulation pathway. Extracted temporal association rules with every parameter set are validated with KEGG cell cycle regulation information. The precision scores of parameter sets are summarized in Table 2. To determine the best parameter set, extracted rules with several sets of parameters, which show relatively high precision scores are examined (precision scores with 0.25, 0.28, and 0.38). The temporal association rules extracted with three selected parameter sets are listed in Figure 3. Finally, [threshold = ± 0.8, support ≥ 3, confidence ≥ 90%] set is selected as the fitted parameter set which shows the highest precision score (0.38). Although the precision score of the fitted parameter set seems not significant, the score is satisfactory in the case of microarray analysis. Because it is reported that when inferring linkages of regulatory proteins in KEGG pathway only from microarray gene expression data set, the accuracy of inferred results were not high owing to the property of microarray itself [30]. Furthermore, we compared the results with Dynamic Bayesian Network (DBN) and Bayesian Network (BN) inference methods. We used the 'G1DBN' package implemented in R for DBN, and we used the 'deal' package implemented in R for BN inference. The result of DBN is optimized for the precision score after exploring possible combinations of parameters. The precision and recall scores of BN are obtained after model averaging. The results of proposed method, DBN, and BN are summarized in Table 3. When comparing precision scores, the proposed method achieved the best performance. However, the proposed method still shows poor recall score like recall scores from two previous methods.

**Table 2:** A summary of precision scores of 70 different parameter sets.

**Confidence**	**90%, 100%**					**80%**				
**Support**	**2**	**3**	**4**	**5**	**6**	**2**	**3**	**4**	**5**	**6**

± 0.2	0.05	0.05	0.06	0.08	0.10	0.05	0.06	0.06	0.07	0.08
± 0.4	0.04	0.06	0.08	0.10	0.15	0.05	0.07	0.08	0.08	0.11
± 0.6	0.05	0.14	0.16	0.17	0.0	0.06	0.14	0.15	0.15	0.13
± 0.8	0.17	0.38	0.25	-	-	0.15	0.25	0.23	0.0	-
± 1.0	0.28	0.0	-	-	-	0.17	0.28	0.0	-	-
± 1.2	0.18	0.0	-	-	-	0.18	0.0	-	-	-
± 1.4	0.0	-	-	-	-	0.0	-	-	-	-

**Table 3:** A summary of precision and recall scores of three methods.

	**TARM**	**DBN**	**BN**
Precision	7/18 = 0.38	3/66 = 0.045	8/50 = 0.16
Recall	7/99 = 0.070	3/99 = 0.030	8/99 = 0.080

### Extracted temporal association rules with fitted parameters

**Figure 4:**
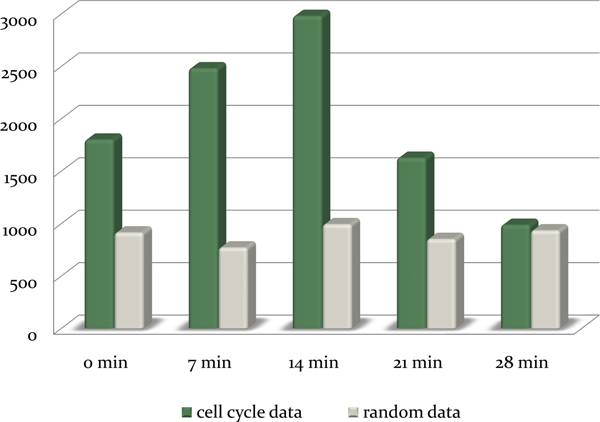
**The number of extracted temporal association rules from cell cycle data set and random data set**. The graph shows the number of extracted temporal association rules in five transcriptional time delays (0, 7, 14, 21, 28 minutes) from time-series gene expression of 799 cell cycle relevant genes and random shuffled cell cycle data set [threshold = ± 0.8, support ≥ 3 transactions, confidence ≥ 90%]. Black bar indicates the number of extracted rules in real data set and gray bar stands for the average number of extracted rules of 100 times of random tests.

Using the selected parameter set, we applied TARM method to 799 genes which are pre-identified as cell cycle relevant genes in [29] and extracted numbers of temporal association rules with various sizes of transcriptional time delay. To test the significance of the temporal association rules, TARM is also applied to random shuffled cell cycle expression data of 799 genes. Figure 4 is the comparison result of both the real cell cycle data set and the shuffled cell cycle data set. As the Figure shows, the extracted numbers of rules from real cell cycle data set and random data set are comparably different. The results indicate that temporal association rules extracted by our proposed method are more significant than random rules.

**Figure 5:**
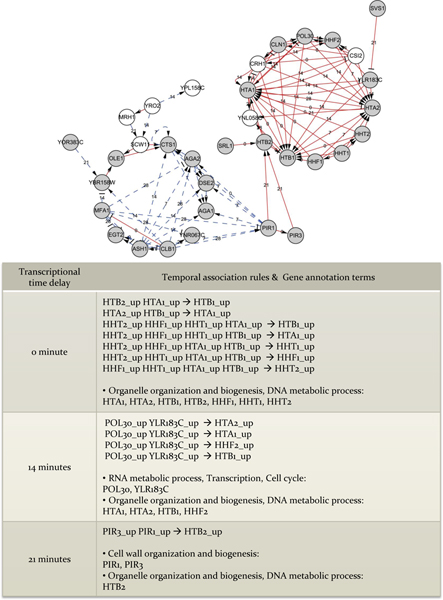
**Validation of the extracted temporal association rules**. Extracted temporal association rules with high support (support ≥ 5) are represented in network structure (upper). A solid pointed arrow edge indicates 'up → up' relation; a solid blunt arrow indicates 'down → up'; a dashed pointed arrow indicates 'down → down'; a dashed blunt arrow indicates 'up → down' relation. Nodes in grey denote genes whose biological function is known. Nodes in white stand for genes whose biological function is not discovered yet. The numeric value on each edge stands for transcriptional time delay (Δ) between genes. Biological process annotation terms of genes represented in network are summarized in Table.

From the extracted temporal association rules, rules with significant support (S ≥ 5) are chosen for further Gene Ontology (GO) term [31] analysis and represented in a directed graph structure (Figure 5). By this analysis, interesting features are found. First, associated genes, which play same role of biological phase with relatively short transcriptional time delay are identified. For example, *HTB2*, *HTA2*, *HHF1*, *HHT1*, *HTB1*, *HTA1*, *HHF2*, and *HHT2 *those who share same annotation term (Organelle organization and biogenesis, DNA metabolic process) are complexly associated with one another within 0~7 minutes and these associated genes are known as having protein interactions with each other. *HTA1 *interacts with *HTA2 *[32], HTB1 [33], *HTB2 *[34,35], *HFF1*[33], *HHT1 *[34-36]. *HTA2 *interacts with *HHF1 *[37], *HHT1*[32], *HHT2 *[32], *HTA1 *[32], *HHF2 *[32]. Second, some temporal dependencies between genes with specific biological processes are detected. Like *POL30*, *YLR183C *(RNA metabolic process, Transcription, Cell cycle) and *HTA1*, *HTA2*, *HTB1*, *HHF2 *(Organelle organization and biogenesis, DNA metabolic process) have temporal association with Δ = 14 minutes. *PIR1*, *PIR3 *(Cell wall organization and biogenesis) and *HTB2 *(Organelle organization and biogenesis, DNA metabolic process) are temporally associated with Δ = 21 minutes.

## Conclusion

We developed the TARM method that can extract temporal association rules in time-series gene expression data, and validated the proposed method with yeast cell cycle gene expression data set. A temporal association rule can describe how the expression of one gene might be associated with the expression of other genes with the related temporal dependency.

In the parameter fitting phase, the best parameter set (threshold = ± 0.8, support ≥ 3 transactions, confidence ≥ 90%), which extracted the most number of correct associations in KEGG cell cycle pathway among 70 combinations of parameters, has been chosen for rule mining. Furthermore, when comparing the precision scores between TARM (0.38), Dynamic Bayesian network (0.045) and Bayesian network (0.16), TARM method showed the best performance. With the best parameter set, numbers of temporal association rules are extracted among pre-identified 799 cell cycle relevant genes. From the extracted temporal association rules, temporally associated genes, which play same role of biological processes (*Organelle organization and biogenesis*, *DNA metabolic process*) with short transcriptional time delay, and some temporal dependencies between genes with specific biological processes are detected. The strong points of our method are the detection abilities of (1) various sizes of transcriptional time delay between associated genes, (2) activation and inhibition relationship, (3) sets of co-regulators for the target genes.

## Competing interests

The authors declare that they have no competing interests.

## Authors' contributions

HN designed the study, implemented the application, performed experiments, and wrote the manuscript. KL participated in the design of the study and performed the result analysis. DL conceived of the study, and participated in its design and coordination and helped to draft the manuscript. All authors read and approved the final manuscript.
